# TMEM106A mediates atherosclerosis progression through macrophage-centered immune responses and chemokine signaling

**DOI:** 10.3389/fimmu.2025.1681645

**Published:** 2025-12-12

**Authors:** Menglong Gao, Xingbang Liu, Zhen Fang, Jia Lun, Qingyun Zhang, Yunbo Zhao, Yin Huang, Zhenhua Li

**Affiliations:** 1 Department of Oncology, WeiFang People’s Hospital, Shandong Second Medical University, Weifang, Shandong, China; 2 Department of Cardiology, Heze Municipal Hospital, Heze, Shandong, China; 3 Department of Cardiology, WeiFang People’s Hospital, Shandong Second Medical University, Weifang, Shandong, China; 4 Phoenix International Medical Center, The Fifth Affiliated Hospital of Sun Yat-sen University, Zhuhai, Guangdong, China

**Keywords:** atherosclerosis, TMEM106A, macrophages, chemokine signaling pathway, PLCB2

## Abstract

**Background:**

Atherosclerosis (AS) remains a leading cause of cardiovascular morbidity and mortality, characterized by intricate interactions between immune dysregulation and lipid metabolism abnormalities—identifying key mediators in its pathogenesis is critical for improving diagnostics and therapies. This study focuses on Transmembrane Protein 106A (TMEM106A) to clarify its role and clinical relevance in AS progression.

**Methods:**

Public transcriptomic datasets (GSE43292, GSE100927, GSE28829) were analyzed to assess TMEM106A expression and diagnostic value; single-cell RNA-seq data (GSE159677) defined its cellular localization. Immune infiltration (ssGSEA, Cibersort, xCell) and CellChat (intercellular communication) analyses explored its immune associations. *In vivo* validation used high-fat diet-induced AS in ApoE^−^/^−^ mice, and *in vitro* experiments with RAW264.7 macrophages included TMEM106A silencing to test functional effects.

**Results:**

TMEM106A was significantly upregulated in AS samples across datasets, with strong diagnostic efficacy (AUC 0.80–0.95). Single-cell analysis confirmed its specific enrichment in macrophages, with functional links to immune-related pathways. TMEM106A promoted macrophage infiltration, foam cell formation, oxidative stress, and inflammatory responses, while regulating PLCB2 in chemokine signaling; silencing TMEM106A alleviated these pro-atherosclerotic effects.

**Conclusion:**

TMEM106A contributes to AS progression by modulating macrophage-mediated immune responses and chemokine signaling, as validated in experimental models. These findings support its potential as a clinically relevant biomarker and promising therapeutic target for AS intervention.

## 1 Introduction

The initiation and progression of atherosclerosis (AS) are governed by a complex interplay of various factors, with the immune system’s modulatory and responsive actions playing a pivotal role. Among these, the involvement of macrophages is notably critical to the etiology of AS (1). Recent advancements in biomedicine have enriched our understanding of how the immune system specifically influences the development of AS. Researchers identified a spectrum of immunological biomarkers, highlighting their prospective utility in the context of the disease. These biomarkers encompass highly sensitive C-reactive protein, which serves as an indicator of acute inflammation; Various interleukins (such as IL-1, IL-6, IL-18) that play a role in mediating immune responses (2); Tumor necrosis factor-alpha (TNF-α), responsible for coordinating the inflammatory environment (3); Monocyte chemoattractant protein-1 that governs leukocyte trafficking (4); And apolipoprotein E (ApoE), a critical lipid and immune modulator (5). These markers provide insights into the immunopathology underlying AS. Beyond their significance in elucidating the mechanisms of AS, these biomarkers hold promise for clinical diagnostics, prognostic assessments, and monitoring therapeutic efficacy (6). However, the heterogeneity and complexity of these markers necessitate careful assessment and judicious interpretation for clinical application. Future investigations aim to seamlessly incorporate such biomarkers into personalized therapeutic strategies, striving to improve patient outcomes and prognoses. However, the specific mechanisms linking immune markers to AS progression remain unclear. Thus, identifying precise biomarkers for AS immunoregulation is of paramount importance.

Transmembrane Protein 106A (TMEM106A) is a pivotal transmembrane biomolecule implicated in a myriad of cellular processes including lipid metabolism, as well as the regulation of cell growth, differentiation, and programmed cell death. This protein plays a critical role in macrophage biology, modulating the expression of key pro-inflammatory mediators like TNF-α and Chemokine (C-C motif) ligand 2 (CCL2), which are essential in the body’s immune responses (7). In the context of AS, cytokines and chemokines like CX3CL1, CCL5, and CXCL8 are crucial to the disease’s pathogenesis, driving immune cell migration and contributing to plaque inflammation (8). Given its regulatory role in chemokine activity, TMEM106A emerges as a promising AS biomarker and a potential target for investigating the disease’s progression through the chemokine signaling pathway.

Macrophages, integral to the innate immune defense, emerge as pivotal contributors to atherogenesis (9). They travel through the bloodstream to arterial plaques, where they engulf oxidized low-density lipoprotein (oxLDL) particles and transform into foam cells. During this process, macrophages generate high levels of reactive oxygen species (ROS), which contributes to plaque growth and impacts plaque stability (10, 11). As AS progresses, an increased presence of macrophages can lead to the development of necrotic cores within plaques, potentially precipitating plaque rupture and acute cardiovascular incidents, including myocardial infarction or stroke (12). Moreover, macrophages secrete pro-inflammatory cytokines and metalloproteinases, which further damage the vascular wall and accelerate the progression of the disease (13). Consequently, strategies to modulate macrophage activity, including the reduction of inflammation, lipid accumulation, and oxidative stress, as well as enhancing their reparative functions, are under investigation to improve plaque integrity and prevent rupture (14, 15). Therefore, searching for targets for macrophage immune infiltration may be the key to treating AS.

Recent studies suggest that TMEM106A is upregulated in murine macrophages, facilitating their polarization towards the M1 phenotype (16). However, it remains unclear whether TMEM106A exhibits similar upregulation in macrophages within atherosclerotic plaques. Therefore, further exploration into how TMEM106A affects the development of AS by regulating macrophage immune infiltration is warranted.

In our study, we employed the robust method of Weighted Gene Co-expression Network Analysis (WGCNA) to pinpoint modules associated with disease pathology. Subsequently, we delved into the intricate network of gene correlations within these identified modules by leveraging advanced analytical techniques, including Random Forest (RF) classification, Lasso regression, and Support Vector Machine Recursive Feature Elimination (SVM-RFE), to enhance our understanding of the genetic underpinnings of the disease. Finally, we used Cibersort, xCell, and ssGSEA immune infiltration algorithms to analyze the correlation between biomarkers and immune infiltrating cells.

## 2 Materials and methods

### 2.1 Analysis and processing of datasets

The initial phase of our research involved the acquisition of public datasets related to AS from the Gene Expression Omnibus (GEO) database. In this process, we gathered a comprehensive suite of four datasets, which included three sets of bulk RNA sequencing data [GSE43292 (GPL6244, Normal: 32, AS: 32) (17), GSE100927 (GPL17077, Normal: 35, AS: 69) (18), GSE28829 (GPL570, Normal: 13, AS: 16) (19)] and one single-cell sequencing GSE159677 (GPL18573, Normal: 3, AS: 3) (20). For the differential expression analysis of datasets GSE43292, GSE100927, and GSE28829, we utilized GEO2R. To identify differentially expressed genes (DEGs), we established rigorous selection criteria, requiring an adjusted P-value (adj.P.Val) below 0.05 and an absolute log2 fold change (|log2 FC|) exceeding 0.5. Comprehensive details pertaining to the datasets are delineated in the **Supplementary Material**, specifically within **Table 1**.

**Table 1:** The information of datasets.

GEO accession	Platforms	Samples (normal)	Samples (AS)	Tissue	Organism	Experiment type
GSE43292	GPL6244/Affymetrix	32	32	Cervical aortic plaque	Homo sapiens	Expression profiling by array
GSE28829	GPL570/Affymetrix	13	16	Cervical aortic plaque	Homo sapiens	Expression profiling by array
GSE100927	GPL17077/Agilent Technologies	49	55	Cervical aortic plaque	Homo sapiens	Expression profiling by array
GSE159677	GPL18573/Illumina	3	3	Cervical aortic plaque	Homo sapiens	Expression profiling by high throughput sequencing

To ascertain marker genes, we leveraged the CellMark database, which is accessible at http://117.50.127.228/CellMarker/ (21). Furthermore, the analysis of biomarker expression within immune cells, incorporating both transcriptional and single-cell data, was facilitated through the utilization of the Human Protein Atlas database, available at https://www.proteinatlas.org/ (22, 23).

### 2.2 Biomarker screening

In our research, we employed the ‘WGCNA’ package within the R programming environment to classify gene modules in the GSE43292 dataset, aiming to identify modules closely associated with atherosclerosis (24). We initiated the analysis with sample clustering to detect and exclude any significant outliers, ensuring robustness in subsequent steps. Following outlier exclusion, we utilized the automated network construction feature within WGCNA to generate a co-expression network, for which we determined an optimal soft-thresholding power (β) to emphasize strong correlations and dampen weaker ones. Module detection was carried out using hierarchical clustering paired with dynamic tree cutting methods. In order to assess the correlation between gene modules and the clinical traits of interest, we computed Gene Significance (GS) and Module Membership (MM) scores. Modules that showed the highest correlation with disease traits were prioritized, and their constituent genes were selected for in-depth analysis in the context of disease relevance.

We constructed a Support Vector Machine (SVM) classifier utilizing the “e1071” package in R and implemented cross-validation to enhance the precision of the regression features (25). Subsequently, we conducted a random forest analysis using the ‘RandomForest’ function to leverage its powerful statistical capabilities. The ‘mtry’ parameter was tuned to correspond with the minimal error rate, and the ‘ntree’ parameter was selected at the juncture where the error rate plateaued. We identified the top 20 pivotal genes by evaluating their feature importance, which was quantified using two metrics: the mean decrease in accuracy (MDA) and the mean decrease in Gini coefficient (MDG) (26). In the final phase of our analysis, we utilized the ‘glmnet’ R package to carry out LASSO regression, which incorporates a regularization penalty to the conventional least squares estimation method, thereby facilitating feature selection through shrinkage of coefficients (27). In performing the LASSO regression analysis, we set the tuning parameters to an alpha of 1 for a pure LASSO model, and the number of lambda values to consider during optimization was specified as 1,000. The value of lambda that yielded the minimum error, lambda.min, was selected as the optimal regularization parameter. The key genes related to the disease were identified by overlapping the gene lists derived from these three machine learning methods.

For WGCNA, key parameters included sample outlier exclusion with a height threshold of 60 (to filter outliers via sample hierarchical clustering), a soft-thresholding power (β) of 30 (determined by the “scale independence” criterion for robust co-expression network construction), module detection via hierarchical clustering combined with dynamic tree cutting (using `minModuleSize = 30` for the minimum number of genes per module, `deepSplit = 2` for moderate module splitting to avoid over-clustering, and `mergeCutHeight = 0.25` to merge modules with a correlation < 0.75 and reduce redundancy), and module-trait correlation thresholds of |Gene Significance (GS)| > 0.2 and |Module Membership (MM)| > 0.8 (to identify modules closely associated with AS traits); for LASSO regression, parameters specified a pure LASSO model (α = 1, distinguishing it from ridge regression or elastic net), 10-fold stratified repeated cross-validation (CV) with 3 repetitions (to ensure balanced AS/normal sample distribution across folds), optimal lambda (λ) selection as `lambda.min` (the λ value minimizing CV error), and retention of genes with non-zero coefficients post-regularization (as candidate biomarkers); for SVM-RFE, the base SVM model used a linear kernel (selected for the lowest CV error after testing radial and polynomial kernels) with a regularization strength (cost parameter C) of 1, the recursive feature elimination process removed 5% of the lowest-ranked features per iteration (stopping when either 10% of original features remained or CV accuracy plateaued), and 5-fold nested CV (with 3 repetitions, including outer folds for model evaluation and inner folds for feature ranking) was applied to select candidate genes; for Random Forest, parameters included 500 decision trees (`ntree = 500`, chosen as CV error plateaued at ~400 trees), 5 variables sampled per split (`mtry = 5`, optimized via 10-fold CV to minimize out-of-bag (OOB) error), prioritization of genes with Mean Decrease in Accuracy (MDA) > 0.05 and Mean Decrease in Gini (MDG) > 1 (as feature importance metrics), and 10-fold repeated CV (3 repetitions) to assess model stability (mean OOB error = 0.11 ± 0.02). Additionally, to eliminate stochastic variability in computational steps, all processes involving randomness—including dataset splitting for LASSO/SVM-RFE/RF cross-validation, Random Forest model initialization, and sample shuffling in WGCNA clustering—were executed with a fixed random seed using R’s `set.seed(123)` function, which was initialized once at the start of all biomarker screening scripts to ensure consistent and replicable results across independent analyses.

### 2.3 ScRNA-seq data analysis

We performed single-cell RNA sequencing data analysis using the Seurat V5 package (28). Initial data quality control was stringent: only cells expressing more than 200 but fewer than 2,500 genes were retained, and cells with mitochondrial gene expression over 5% were excluded. We then normalized and scaled the gene expression matrices for the high-quality cells with the ‘NormalizeData’ and ‘ScaleData’ functions, respectively, to facilitate linear transformation of the data. For dimensionality reduction, we selected the top 2,000 highly variable genes and carried out principal component analysis (PCA), utilizing the 20 most significant principal components for subsequent clustering. To mitigate batch effects from merging datasets from multiple samples, we applied the ‘Runharmony’ function from the Harmony package (29). Visualization of the clusters was achieved using the UMAP technique. Finally, we annotated the cell types by their expression profiles of known marker genes.

### 2.4 Animal administration

Protocols involving animal subjects were conducted in strict compliance with ethical standards, having received full approval from the Animal Care and Use Committee at Shandong Second Medical University (Approval date: Nov 18, 2022; Approval Number: 2022SDL322). Estrogen can significantly influence the development of atherosclerosis. To eliminate the variable effects of age-related changes in estrogen levels. Specifically, Male ApoE^-/-^ mice were used in the present study and maintained in a sterile environment. The vivarium was set with a controlled 12-hour light-dark cycle to mimic natural conditions. To promote wellbeing and natural behavior, mice were housed in ventilated plastic enclosures measuring 30 cm x 20 cm x 20 cm, with a maximum of three mice per cage to prevent overcrowding.

Throughout the study, the male ApoE^-/-^ mice enjoyed unrestricted access to standard rodent feed and water. Following a seven-day acclimatization period, they were placed on a high-fat diet (HFD) comprising 21% fat and 0.15% cholesterol for a duration of 12 weeks to instigate the formation of atherosclerotic lesions. Male C57BL/6 mice fed a regular diet were used as a control group.

### 2.5 Cell culture and treatments

The RAW264.7 cell line used in this study was sourced from the Cell Resource Center of the Shanghai Institute for Biological Sciences, Chinese Academy of Sciences. These cells were cultured in Dulbecco’s Modified Eagle Medium (DMEM) containing 10% fetal bovine serum, with maintenance in a humidified incubator set to 37°C and 5% CO_2_. To guarantee experimental consistency and preserve stable cellular traits, only cells from passages 4 to 8 were utilized for all assays in this research.

### 2.6 RNA interference targeting TMEM106A and transfection procedure

For TMEM106A knockdown in RAW264.7 macrophages, three specific small interfering RNAs (siRNAs) with sequences 5’-GCUAGUUCCAGCUUUGUGATT-3’, 5’-CGACGAAUGUCCUGAACAUTT-3’, and 5’-GUUGCUCUCAUCCCUUAUGTT-3’ (targeting human TMEM106A, GenBank: NM_024757) and a non-targeting scramble siRNA (negative control) were used; all siRNAs were chemically synthesized by GenePharma Co., Ltd. (Shanghai, China; TMEM106A siRNA pool: GP-10624; negative control: GP-001) with >95% purity and no mouse/human genome homology to avoid off-target effects. Transfections were performed using the Lipofectamine 3000 Transfection Kit (Invitrogen, L3000015) per the manufacturer’s RAW264.7-optimized protocol: RAW264.7 cells were seeded in 6-well plates at 4×10^5^ cells/well, cultured in high-glucose DMEM (Gibco, 11965092) with 10% FBS (Gibco, 10099-141) at 37°C and 5% CO_2_ until 80% confluency, then 5 μL of siRNA pool (50 nM final, equal siRNA-1/2/3 mix) or negative control siRNA was mixed with 150 μL Opti-MEM I (Invitrogen, 31985070) and combined with 5 μL Lipofectamine 3000 in 150 μL Opti-MEM I; the mixture was incubated 5 minutes at room temperature to form complexes, which were added dropwise to cells for continuous culture at 37°C and 5% CO_2_.

Under the same 37°C and 5% CO_2_ incubation conditions, RAW264.7 cells (untransfected or siRNA-transfected) were subjected to functional induction: 80 μg/mL oxidized low-density lipoprotein (oxLDL; MedChemExpress, catalog number: HY-NP135) for 24 hours to induce macrophage foam cells, and 1 μg/mL lipopolysaccharide (LPS; Sigma-Aldrich, L2630) for 24 hours to induce macrophage inflammation; induced cells were used for subsequent assays.

### 2.7 ELISA analysis

RAW264.7 cells were seeded in a 24-well plate at a density of 1 × 10^5^ cells per well in 500 μL of medium and allowed to adhere overnight. The following day, the cells were treated with lipopolysaccharide and oxidized low-density lipoprotein for a 24-hour incubation period. Upon completion of the treatments, we harvested the culture supernatants for the quantification of cytokine levels. Concentrations of TNF-α (JL10484-48T), IL-1β (JL20884-48T), and IL-6 (JL33712-48T) were measured using ELISA kits from Jianglai Biotechnology, Shanghai, China, strictly adhering to the manufacturer’s protocols. The resultant absorbances were read and recorded using a microplate reader for subsequent analysis.

### 2.8 Real-time quantitative PCR

Total RNA was extracted utilizing the Total RNA Isolation Reagent provided by Biosharp, Anhui, China. This RNA served as the template for cDNA synthesis, which was performed using a reverse transcription kit from SparkJade Science, Shandong, China. The gene-specific primers for GAPDH were as follows: forward 5’-GAGAGTGTTTCCTCGTCCCG-3’ and reverse 5’-ATCCGTTCACACCGACCTTC-3’. TMEM106A primers were: forward 5’-AGCTCACCTCTCGGAAGGAT-3’ and reverse 5’-ATTGCCTTGGCAGGTAGGAC-3’, while PLCB2 primers were: forward 5’-TGGAGTTCCTGGATGTCACG-3’ and reverse 5’-GTGAGGTCCACCATGTCAGG-3’. Parallel amplification of GAPDH served as an internal control to normalize the data. Relative gene expression levels were quantified employing the 2^-ΔΔCt^ method.

### 2.9 Immunofluorescence staining

Samples were fixed in 4% paraformaldehyde (PFA) for 15 minutes at room temperature to preserve cellular structure. After permeabilization with 0.1% Triton X-100 in PBS, the samples were blocked with 0.5% bovine serum albumin (BSA) dissolved in PBS to minimize non-specific antibody binding. Primary antibodies against F4/80 (Abcam, ab6640) and TMEM106A (Abcam, ab140192) were incubated overnight at 4 °C. Subsequently, secondary antibodies, including Alexa Fluor 488-conjugated goat anti-rabbit IgG (H+L) (Biyuntian, catalog number A0423, dilution 1:500) and Alexa Fluor 594-conjugated donkey anti-rat IgG (H+L) (Abcam, catalog number ab150156, dilution 1:500), were incubated at room temperature for 2 hours. Nuclei were stained with DAPI (Biyuntian, catalog number C1005) for 15 minutes, and images were captured using a NIKON ECLIPSE 100 fluorescence microscope.

### 2.10 ROS assessment

ROS contents were assessed by fluorescence microscopy using a DCFH-DA oxidationsensitive fluorescent probe (S0033S, Beyotime Biotechnology, Shanghai, China). Detection of ROS content by flow cytometry (S0033S, Beyotime Biotechnology, Shanghai, China).

### 2.11 Functional enrichment analysis

To elucidate the biological functions and involved pathways of the identified genes, Gene Ontology (GO) and Kyoto Encyclopedia of Genes and Genomes (KEGG) pathway enrichment analyses were performed utilizing the ‘clusterProfiler’ package in R. This comprehensive approach allowed for a systematic investigation of the gene sets in the context of their associated biological processes, cellular components, molecular functions, and predefined pathway networks (30–32). GO and KEGG analysis gene in the **Supplementary Table S1**. The results of GO and KEGG are presented in **Supplementary Tables S2** and **S3**. Gene Set Enrichment Analysis (GSEA) was employed to identify alterations in pathways and biological processes within datasets (33). The results of GSEA are presented in **Supplementary Table S4**. Furthermore, we applied the single-sample Gene Set Enrichment Analysis (ssGSEA) algorithm to calculate individual pathway activity scores for each sample, deriving these scores from the specific gene expression data of each sample. This method enables a nuanced assessment of the pathway dynamics unique to each biological specimen (34).

To bolster the robustness of our enrichment analysis, we applied the Benjamini-Hochberg procedure for False Discovery Rate (FDR) correction. This critical step is taken to mitigate the risks of false positives due to multiple hypothesis testing by adjusting the p-values accordingly. An FDR threshold of less than 0.05 was used to denote statistically significant enrichment, thus minimizing the likelihood of false positives due to multiple comparisons.

### 2.12 Immune cell infiltration analysis

Three computational algorithms—single-sample Gene Set Enrichment Analysis (ssGSEA), Cibersort, and xCell—were employed to meticulously evaluate immune cell infiltration. The ssGSEA was executed utilizing the “GSVA” package in R, facilitating the quantification of the infiltration levels for 28 distinct immune cell types, thus providing a comprehensive profile of the immune landscape within the samples (35). While conducting a ssGSEA to screen for the correlation between immune cell populations and TMEM106A expression, we identified and selected three datasets where immune cells demonstrated a robust correlation, with a Pearson correlation coefficient (r) exceeding 0.7. This threshold denotes a robust positive linear correlation among the variables within the selected datasets. Utilizing the “xCell” package in R, the xCell algorithm precisely estimated the proportions of 64 immune cell types, thereby enriching our understanding of the immunological composition in the samples (36). The Cibersort algorithm was employed to accurately calculate the proportions of 22 distinct immune cell types within each sample. This advanced computational method allowed for a precise assessment of the cellular composition across all samples (37). The results of the immune infiltration analysis were visualized using the widely-used “ggplot2” R package, known for its robust plotting capabilities. Utilizing this package, the data obtained from the analysis were transformed into visually appealing visualizations, enabling a comprehensive representation of the results (38). Violin plots were used to showcase the expression levels of immune infiltrating cells across various subgroups. A stacked bar chart was created to present the proportions of immune cells in each sample, derived from the Cibersort analysis. Moreover, correlation heatmaps were generated to demonstrate the Pearson correlations between biomarkers or pathways and immune infiltrating cells.

### 2.13 Statistical analysis

For data processing and statistical analyses, R software (version 4.3.1) and GraphPad Prism (version 9.2.0) were employed; for two-group comparisons, unpaired two-tailed t-tests were used for data with normal distribution and homogeneous variance, while the Mann-Whitney U test (a non-parametric alternative) was applied to variables that deviated from normality, with normality verified by the Shapiro-Wilk test and variance homogeneity confirmed via Levene’s test. For comparisons involving multiple groups with non-normally distributed continuous variables, the Kruskal-Wallis test was utilized, and a p-value less than 0.05 was considered statistically significant, with the specific statistical tests applied for each experiment indicated in the corresponding figure legends.

## 3 Result

### 3.1 AS biomarker screening

To identify potential biomarkers for Atherosclerosis (AS), we integrated three machine learning methods with Weighted Gene Co-expression Network Analysis (WGCNA), and first analyzed the three public datasets (GSE43292, GSE100927, GSE28829) at the single-cell clustering and differential gene level.

For the three datasets, we performed UMAP clustering and differential expression analysis. As shown in **Supplementary Figure S1**, UMAP plots clearly distinguished AS samples from normal samples in each dataset (GSE43292, GSE100927, GSE28829), and volcano plots revealed numerous genes with significant differential expression between AS and normal groups (Padj < 0.05), laying a foundation for subsequent biomarker screening.

**Figure 1:**
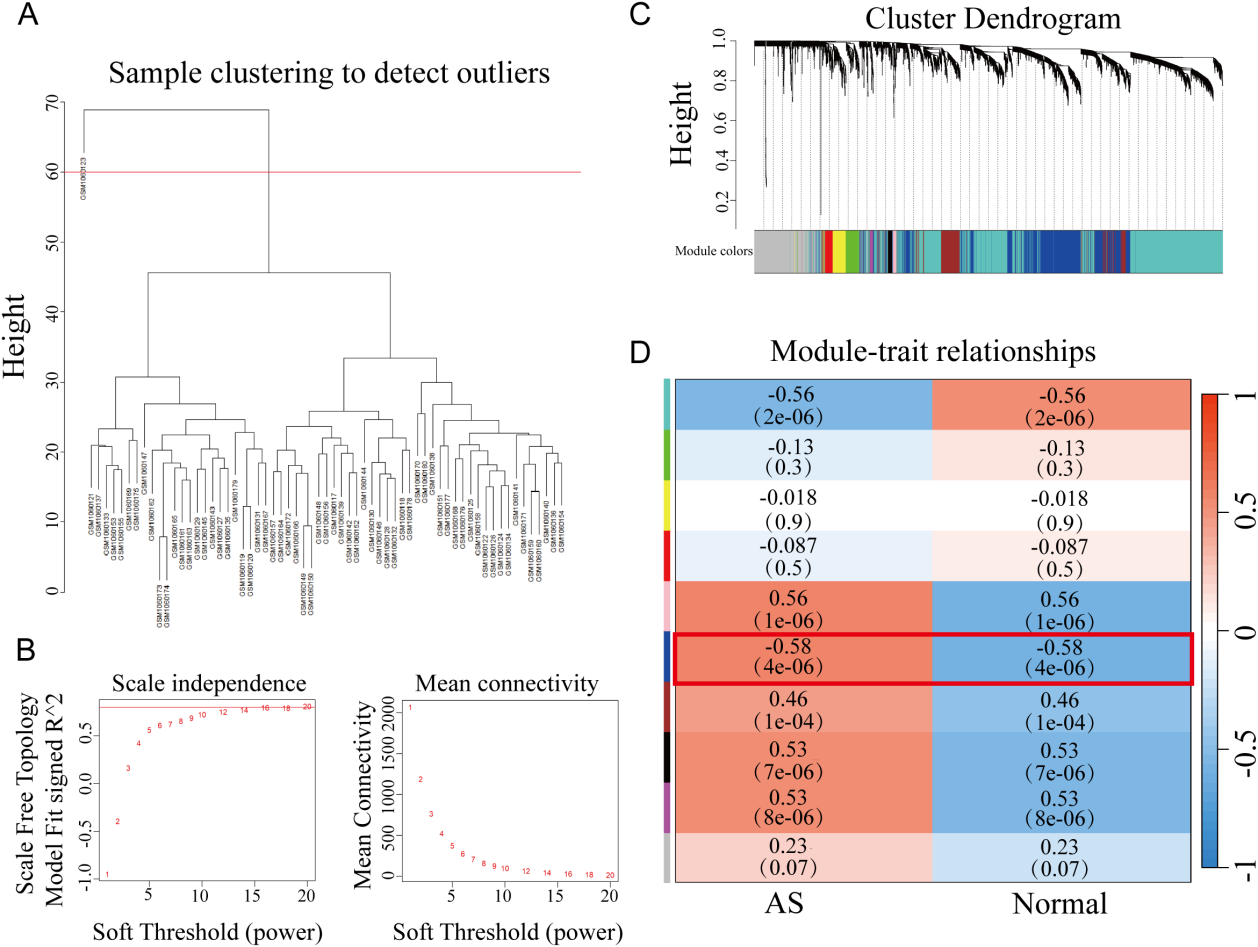
Sample clustering and WGCNA module-trait association. **(A)** Sample clustering dendrogram for outlier detection, with a height threshold of 60. **(B)** Analysis of scale independence and mean connectivity to determine the soft thresholding power (set at 30 based on a correlation coefficient threshold of 0.85). **(C)** Cluster dendrogram of 10 gene modules identified by WGCNA. **(D)** Module-trait relationship heatmap showing the correlation between modules and AS traits, with the blue module exhibiting the highest correlation (r = 0.57, *p* = 4e-07).

In the WGCNA workflow, we first carried out sample clustering to eliminate outliers (setting the height threshold at 60, as depicted in **Figure 1A**). A soft-thresholding power of 30 was chosen, guided by a correlation coefficient threshold of 0.85 (**Figure 1B**). Through WGCNA-based network clustering, genes from the GSE43292 dataset were categorized into 10 distinct modules (**Figure 1C**). We prioritized the blue module, which exhibited the highest relevance to AS (r = 0.57, p = 4e-07, **Figure 1D**), for subsequent machine learning investigations.

**Figure 2:**
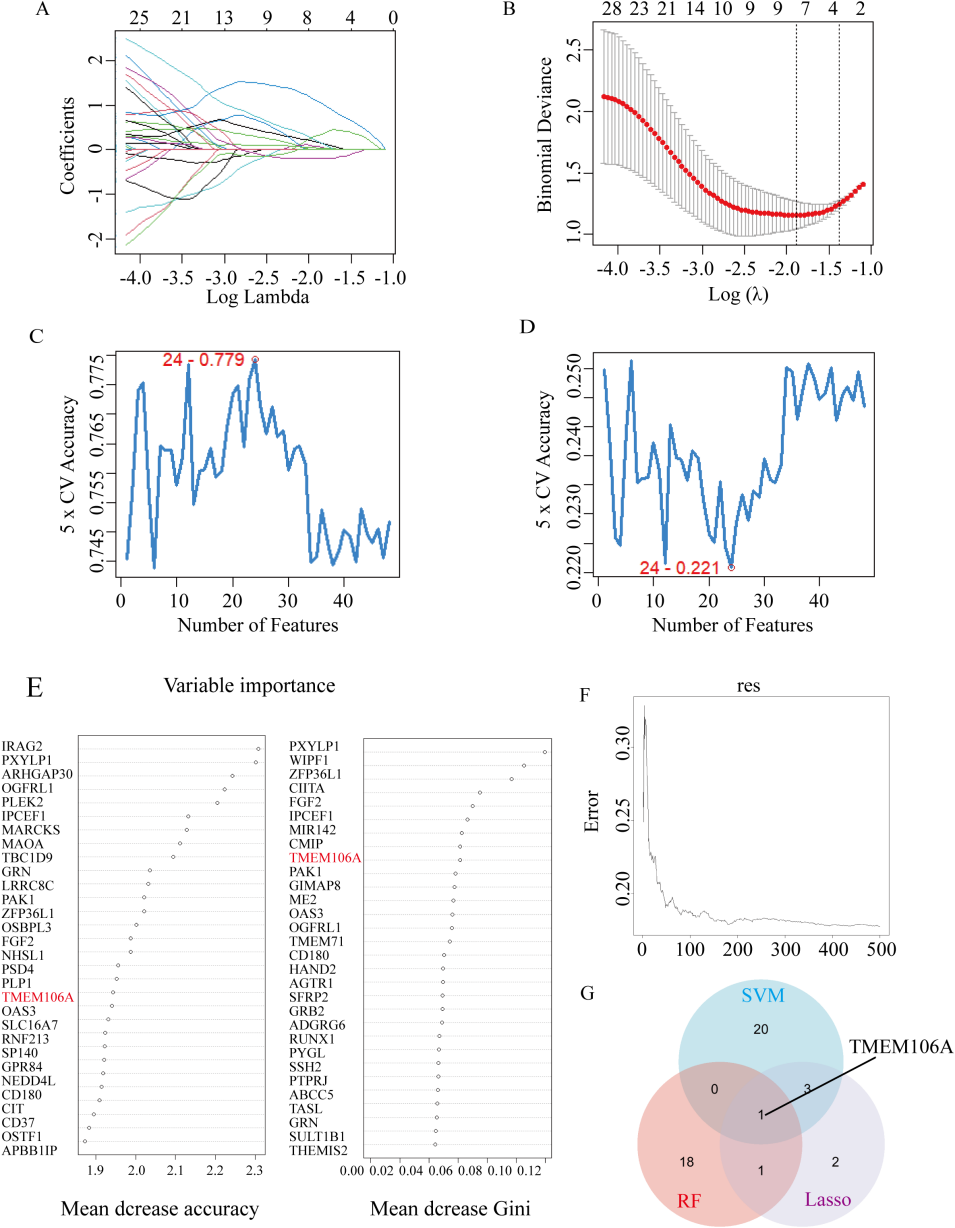
Biomarker screening by machine learning algorithms. **(A, B)** Lasso algorithm results: coefficient distribution and cross-validation plot for identifying 7 potential biomarkers. **(C, D)** SVM-RFE algorithm results: feature importance ranking and cross-validation curve for filter 24 key candidate biomarkers. **(E, F)** Random Forest algorithm results: variable importance (mean decrease accuracy and Gini index) for 20 prospective biomarkers. **(G)** Venn diagram showing the intersection of biomarkers identified by RF, Lasso, and SVM-RFE, with TMEM106A as the unique common biomarker.

Utilizing the Lasso algorithm on this module, 7 potential biomarkers were identified (**Figures 2A, B**). The SVM-RFE (Support Vector Machine-Recursive Feature Elimination) algorithm further filtered out 24 key candidate biomarkers for AS (**Figures 2C, D**). Additionally, the RF (Random Forest) algorithm pinpointed 20 prospective biomarkers (**Figures 2E, F**). Ultimately, across these algorithms, TMEM106A emerged as the unique key biomarker for AS (**Figure 2G**).

### 3.2 TMEM106A is highly expressed in AS

To clarify TMEM106A’s role in AS, we investigated its expression in AS patients and ApoE^-/-^ mice with a high-fat diet (HFD).

**Figure 3:**
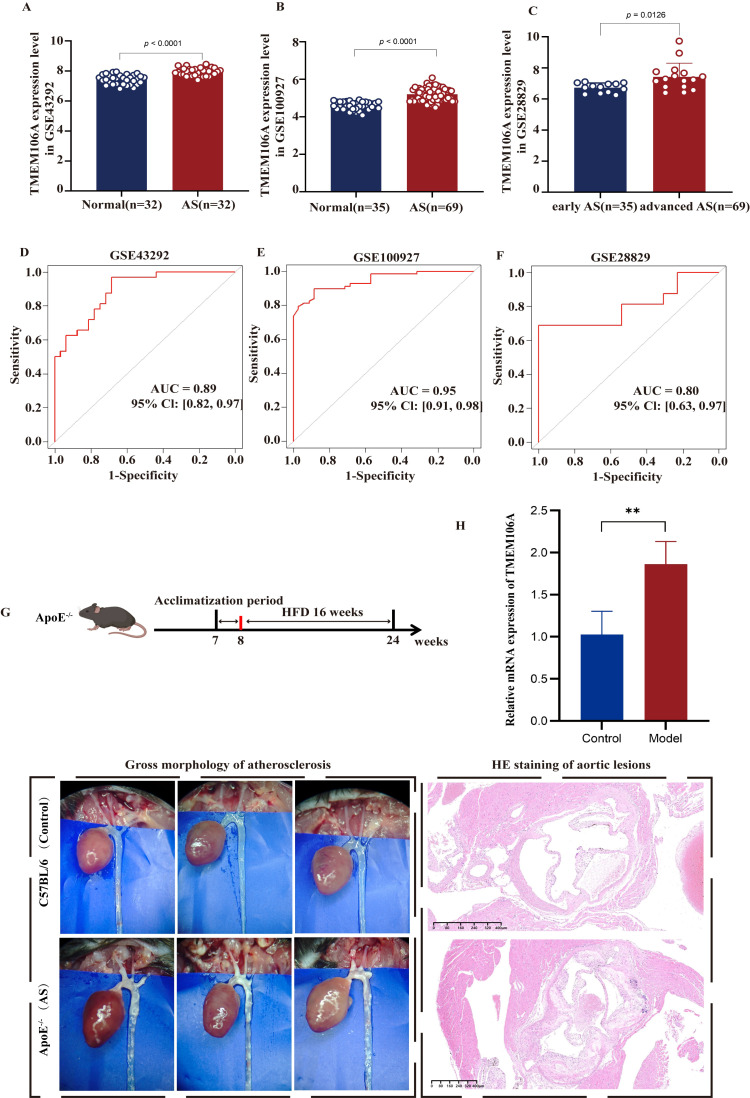
Shows TMEM106A’s role in AS diagnosis and validation in a mouse model. **(A-C)** TMEM106A expression is higher in AS than normal samples across three datasets (GSE43292, GSE100927, GSE28829), with advanced AS showing elevated levels vs early AS in GSE28829. **(D-F)** ROC curves confirm TMEM106A’s diagnostic value, with AUCs of 0.89 (GSE43292), 0.95 (GSE100927), and 0.80 (GSE28829). **(G)** ApoE^^−^/^−^^ mice on a high-fat diet develop AS, shown by aortic plaques and H&E-stained lesions (Left panels: gross morphology of atherosclerosis; Right panels: HE staining of aortic lesions). **(H)** TMEM106A mRNA (via qPCR) are upregulated in the model group vs controls (*p* < 0.01, n = 6). **p < 0.01.

Leveraging multiple datasets (GSE43292, GSE100927, GSE28829), we first analyzed clinical samples. As shown in **Figures 3A–C**, TMEM106A expression was significantly elevated in AS patients compared to normal controls (all p < 0.05). To evaluate its diagnostic potential, we constructed ROC curves. The results (**Figures 3D–F**) showed AUC values of 0.89, 0.95, and 0.80, reflecting strong discriminatory ability for distinguishing AS samples from normal ones.

We next performed *in vivo* validation using ApoE^^−^/^−^^ mice. Following 16 weeks of high-fat diet (HFD) induction, gross morphological analysis of atherosclerosis and hematoxylin-eosin (HE) staining of aortic lesions (**Figure 3G**) were conducted, and subsequent RT-qPCR analyses (**Figure 3H**) confirmed that aortic TMEM106A expression was markedly higher in HFD-fed ApoE^^−^/^−^^ mice than in normal C57BL/6 mice (both p < 0.05).

Collectively, these multi-level evidence (clinical datasets + animal model) consistently demonstrate that TMEM106A is highly expressed in AS, supporting its potential as a disease-associated biomarker.

### 3.3 TMEM106A links to macrophage-centered immune responses in atherosclerosis

**Figure 4:**
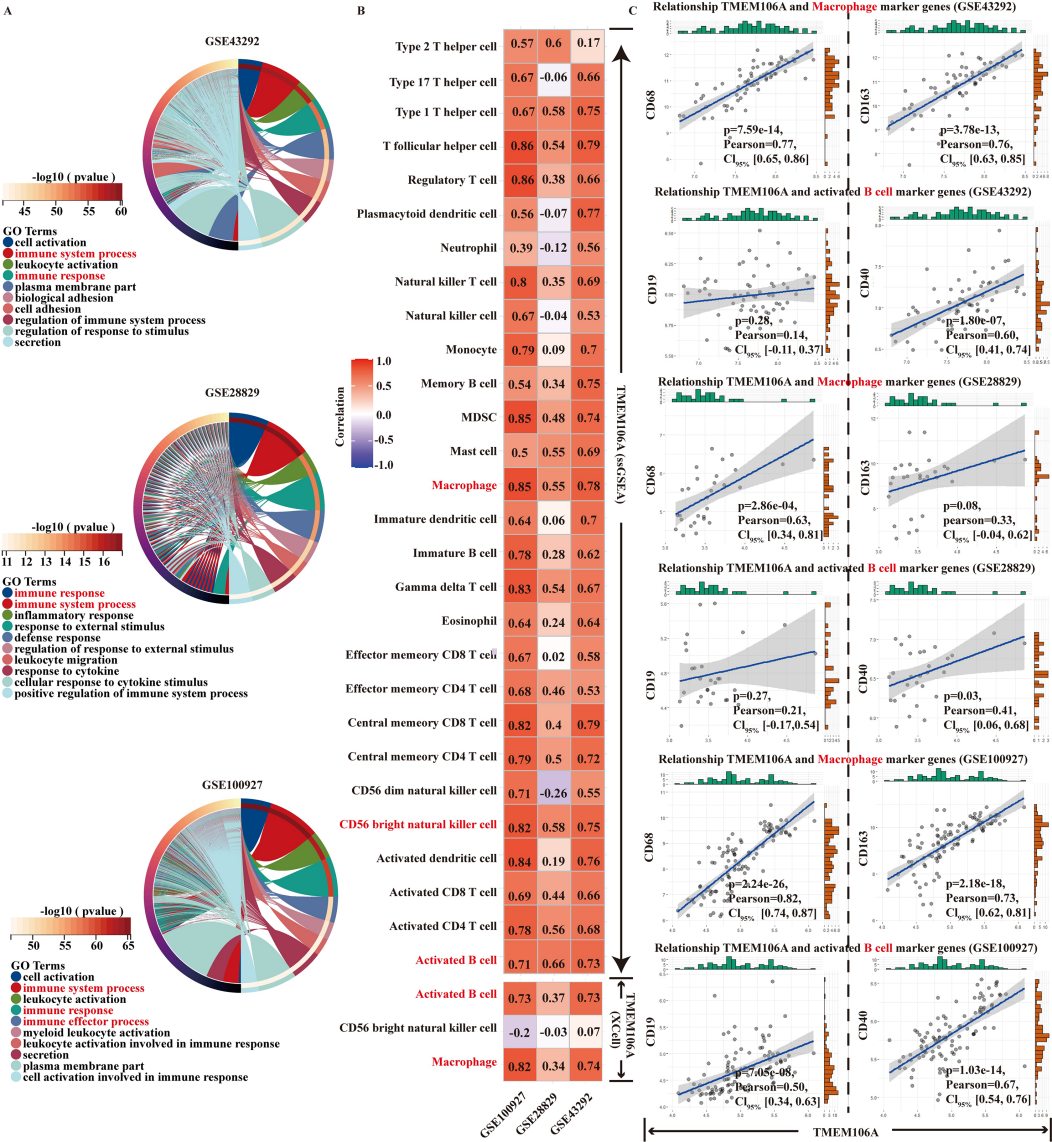
**(A)** GO functional enrichment of TMEM106A-associated genes in GSE43292, GSE28829, GSE100927 (circular plots show enriched immune-related terms). **(B)** Correlation of TMEM106A with immune cell populations (heatmap displays macrophage/activated B cell associations) across datasets. **(C)** Scatter plots of TMEM106A vs macrophage and B cell markers, highlighting stronger macrophage correlations.

To elucidate the immunological relevance of TMEM106A in AS, we performed multi-step analyses integrating functional enrichment, immune infiltration, and correlation assessments. Functional enrichment of three datasets (GSE43292, GSE28829, GSE100927) via GO analysis (**Figure 4A**) revealed that TMEM106A-associated genes were robustly enriched in immune-related signaling pathways and biological processes (e.g., immune response activation, regulation of immune responses to stimuli), implicating TMEM106A in the AS immune regulatory network. Next, immune cell infiltration profiling using ssGSEA (**Supplementary Figure S2**) showed that macrophages exhibited significant infiltration differences across the three datasets, with distinct distribution patterns between AS and normal groups; Cibersort analysis (**Supplementary Figure S3**) further confirmed that macrophages accounted for a large proportion of infiltrating immune cells in all datasets, with their relative percentage notably higher than other cell types such as B cells and T cells; XCell analysis (**Supplementary Figure S4**) validated these findings by demonstrating significant differential infiltration of macrophages between AS and normal samples, with macrophage-related scores markedly elevated in AS, and these multi-tool results consistently indicated significant associations between TMEM106A and infiltration of macrophages and activated B cells in AS samples, with macrophages showing higher enrichment, suggesting TMEM106A may drive macrophage-mediated immune processes in AS pathogenesis while also engaging B cell responses. Finally, correlation analysis of TMEM106A with immune cell marker genes across datasets (**Figure 4C**) uncovered strong positive correlations between TMEM106A and macrophage markers (e.g., CD68, CD163; significant p-values, high Pearson coefficients), which were more prominent than correlations with B cell markers (e.g., CD19, CD20). Collectively, these findings highlight TMEM106A’s tight linkage to macrophage-centered immune responses in the AS microenvironment, supporting its potential as a key mediator of immune-driven AS progression.

### 3.4 Macrophage-specific high expression of TMEM106A validated by scRNA-seq and immunofluorescence

**Figure 5:**
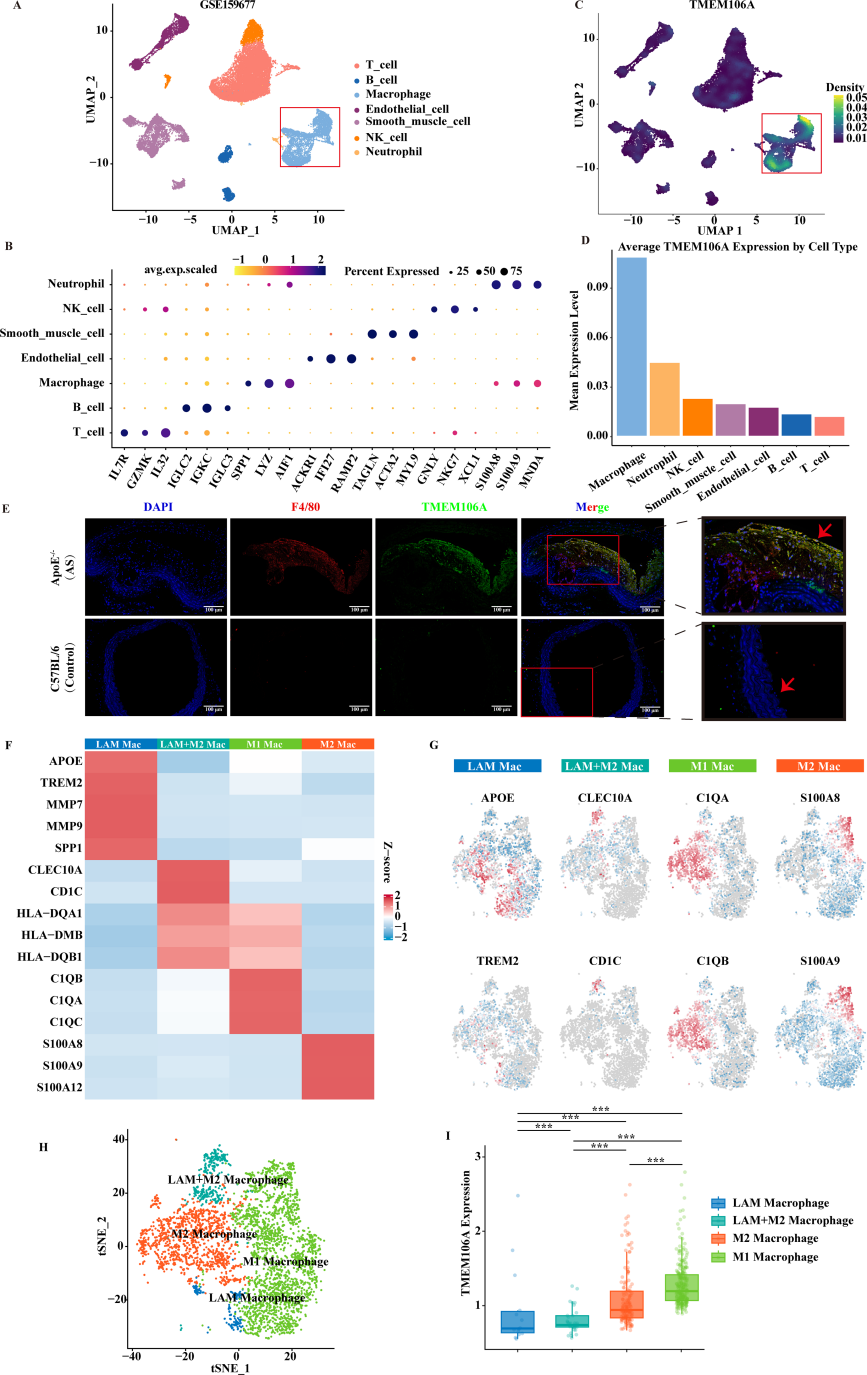
Single-cell and immunofluorescence analysis of TMEM106A in atherosclerotic lesions. **(A)** UMAP clustering of GSE159677 scRNA-seq data, identifying major cell populations (neutrophils, endothelial cells, macrophages, B cells, T cells). **(B)** Heatmap of TMEM106A expression percentage across cell types. **(C)** Average TMEM106A expression per cell type, highest in macrophages. **(D)** UMAP plot of TMEM106A expression, concentrated in the macrophage cluster. **(E)** Immunofluorescence: TMEM106A (green) co-localizes with F4/80 (red) in ApoE^-/-^ mouse aortas (AS model), minimal in C57BL/6 controls (DAPI, blue nuclei, Scale bar = 100 μm). **(F)** Z-score heatmap of marker genes across four macrophage subsets: LAM Mac, LAM+M2 Mac, M1 Mac, M2 Mac. **(G)** tSNE plots of representative marker genes (APOE, CLEC10A, CIQA, S100A8, TREM2, CD1C, C1QB, S100A9) across macrophage subsets. **(H)** tSNE plot of four macrophage subsets, each colored distinctly. **(I)** Violin plot of TMEM106A expression across subsets; ***P < 0.001** (one-way ANOVA with Tukey’s test).

To clarify the cellular localization of TMEM106A in AS, we first performed quality control and preprocessing on single-cell data as shown in **Supplementary Figure S5** (analyzing metrics like `nFeature_RNA`, `nCount_RNA`, and `percent.mt` for cell filtering, followed by normalization, dimensionality reduction, and variable gene selection), and then analyzed the single-cell RNA-seq dataset GSE159677. UMAP clustering (**Figure 5A**) identified major cell populations in atherosclerotic samples, with macrophages highlighted (red box).

Marker gene validation for each cell type in GSE159677 (**Figure 5B**) confirmed cell identity first. Then, TMEM106A expression mapping (**Figure 5C**) showed concentrated signals within the macrophage cluster.

Average expression quantification (**Figure 5D**) revealed that TMEM106A was most highly expressed in macrophages, far exceeding other cell types (e.g., T cells, B cells). To verify this, immunofluorescence double-staining (**Figure 5E**) was performed on ApoE^-/-^ mice (AS model) and control C57BL/6 mice. Co-staining of TMEM106A (green) with the macrophage marker F4/80 (red) showed robust co-localization in ApoE^-/-^ mouse aortas, while control mice exhibited minimal TMEM106A signals.

Among the annotated cell clusters, macrophages were further stratified into four distinct subsets (LAM Macrophage [LAM Mac], LAM+M2 Macrophage [LAM+M2 Mac], M1 Macrophage [M1 Mac], and M2 Macrophage [M2 Mac]) based on differential marker gene expression (**Figure 5H**). A Z-score-normalized heatmap (**Figure 5F**) visualized the expression patterns of subset-specific marker genes: LAM Mac was characterized by high expression of APOE and TREM2; LAM+M2 Mac exhibited enrichment of CLEC10A and CD1C; M1 Mac was marked by CIQA and C1QB; while M2 Mac showed prominent expression of S100A8 and S100A9.

To visualize the distribution of representative marker genes across macrophage subsets, t-distributed stochastic neighbor embedding (tSNE) plots (**Figure 5G**) confirmed that representative genes (e.g., APOE for LAM Mac, CLEC10A for LAM+M2 Mac, CIQA for M1 Mac, S100A8 for M2 Mac) were selectively expressed in their corresponding macrophage subsets, validating the sub-clustering accuracy.

We further quantified TMEM106A expression across these four macrophage subsets using a violin plot (**Figure 5I**). Statistical analysis (one-way ANOVA with Tukey’s multiple comparison test) revealed significant differences in TMEM106A expression among subsets (*P < 0.001): M1 Mac showed the highest TMEM106A expression, followed by M2 Mac, while LAM+M2 Mac and LAM Mac exhibited relatively lower expression levels. These results indicated that TMEM106A is preferentially expressed in pro-inflammatory macrophage subsets (M1) within atherosclerotic lesions.

These results collectively demonstrate that TMEM106A is specifically enriched in macrophages during AS progression, supporting its macrophage-centered functional role.

### 3.5 Cell-cell communication analysis of TMEM106A in AS at the single-cell level

**Figure 6:**
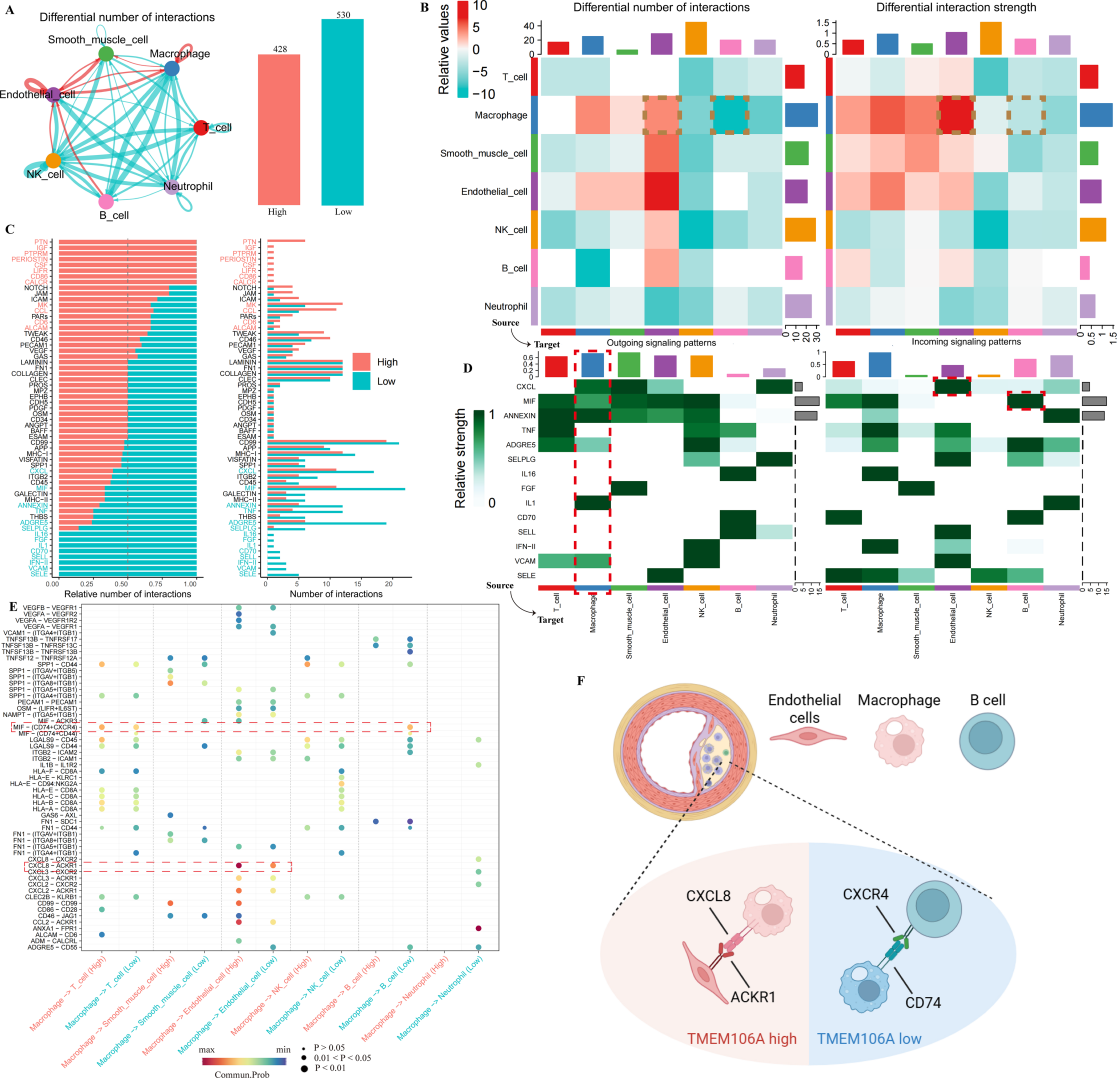
Cell-cell communication of TMEM106A in AS. **(A)** CellChat analysis of GSE159677 shows low-TMEM106A groups have stronger cell-cell interactions. **(B)** Heatmaps display differential interaction numbers/strengths, with prominent macrophage-endothelial & endothelial-B cell crosstalk. **(C)** Bar plots highlight key receptor-ligand pathways (e.g., CXCL, MIF). **(D)** Macrophage ligand-receptor heatmaps show strong CXCL pathway activity. **(E)** Dot plots detail pathway differences between high-vs low-TMEM106A groups. **(F)** Schematic models: High-TMEM106A relies on CXCR4-CD74 (B cell-macrophage); Low-TMEM106A on CXCR8-ACKR1 (macrophage-endothelial).

To further elucidate the role of TMEM106A in Atherosclerosis (AS), we performed cell-cell communication analysis on the single-cell RNA-seq dataset GSE159677 using CellChat, as shown in **Figure 6**.

We first compared the cell communication profiles between the high-and low-TMEM106A expression groups. As depicted in **Figure 6A**, the low-expression group exhibited stronger overall cell-cell interaction strength. In **Figure 6B**, we observed that the communication intensity between macrophages and endothelial cells, as well as between endothelial cells and B cells, was more prominent.

**Figure 6C** displayed the significant receptor-ligand pathways involved in these cell communications. For macrophages (**Figure 6D**), the CXCL ligand-receptor pathway showed stronger emission strength. Regarding endothelial cells, among the received signals, the reception related to the CXCL pathway was the strongest during communication with macrophages. For B cells, the reception of the MIF (Macrophage Migration Inhibitory Factor)-related receptor pathway was the most prominent.

Specifically, from the cell communication model in **Figure 6F**, in the high-TMEM106A group, CXCR4 serves as a receptor on B cells, while CD74 acts as a ligand on macrophages, and their interaction is the most potent. In the low-TMEM106A group, the interaction between CXCR8 (in macrophages) and ACKR1 (in endothelial cells) was the most prominent. These findings collectively illustrate the distinct cell-cell communication patterns associated with different TMEM106A expression levels in AS, highlighting the potential regulatory roles of TMEM106A in immune cell crosstalk within the atherosclerotic microenvironment.

### 3.6 TMEM106A promotes macrophage foam cell formation, oxidative stress, and inflammation

**Figure 7:**
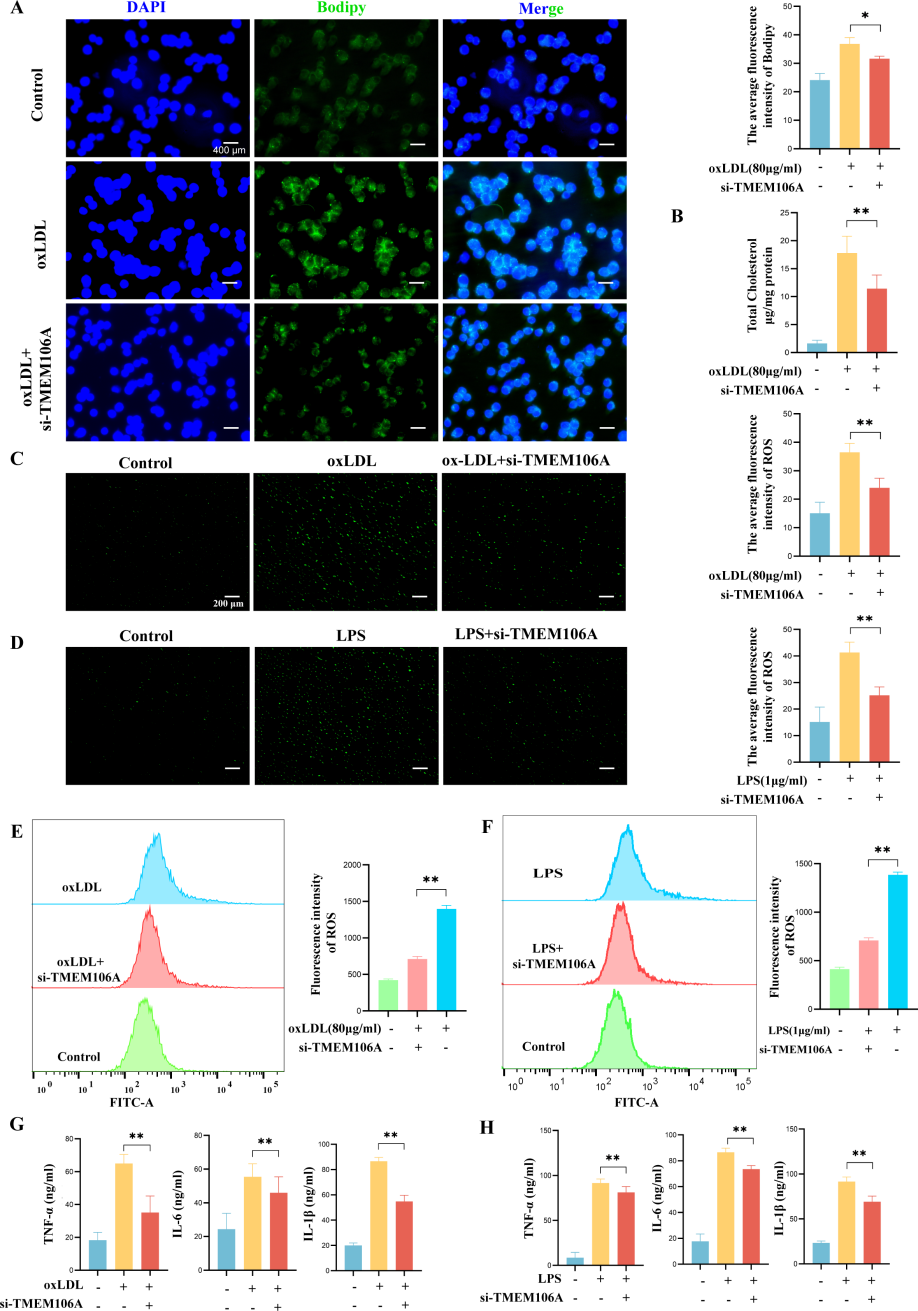
TMEM106A promotes macrophage foam cell formation, oxidative stress, and inflammation. **(A)** Bodipy staining showing reduced lipid accumulation (green fluorescence) in oxLDL (80μg/ml)-treated macrophages after TMEM106A silencing (si-TMEM106A) (n = 3, Scale bar = 400 μm). **(B)** TC assay confirming suppressed total cholesterol elevation in si-TMEM106A-treated macrophages (n = 6). **(C, D)** DCFH-DA staining showing reduced ROS levels (green fluorescence) in oxLDL/LPS-stimulated macrophages with TMEM106A silencing (n = 3, Scale bar = 200 μm). **(E, F)** Flow cytometry (FITC-A) verifying decreased ROS mean fluorescence intensity in si-TMEM106A groups (n = 3). **(G, H)** ELISA results showing inhibited secretion of pro-inflammatory cytokines (TNF-α, IL-1β, IL-6) in si-TMEM106A-treated macrophages under oxLDL/LPS stimulation (n = 6). *p < 0.05, **p < 0.01.

To investigate the functional role of TMEM106A in macrophages during AS progression, we first performed a series of *in vitro* assays. For foam cell formation, Bodipy staining (**Figure 7A**) showed that oxLDL-induced macrophage lipid accumulation was significantly reduced after TMEM106A silencing (si-TMEM106A), as evidenced by decreased green fluorescence intensity. TC kit assays (**Figure 7B**) further confirmed that TMEM106A knockdown suppressed oxLDL-induced total cholesterol elevation in macrophages.

Regarding oxidative stress, we first ensured accurate identification of the target macrophage population through flow cytometry gating (**Supplementary Figure S6**), where we gated on lymphocytes via the FSC-A/SSC-A plot and further analyzed FITC-A signals to minimize interference from other cell types. Based on this, DCFH-DA staining (**Figures 7C, D**) and flow cytometry (**Figures 7E, F**) revealed that both oxLDL and LPS stimulation increased ROS levels in macrophages, while TMEM106A silencing markedly attenuated this oxidative response, as indicated by reduced green fluorescence signals and lower mean fluorescence intensity of ROS.

For inflammation, ELISA assays (**Figures 7G, H**) demonstrated that oxLDL or LPS-induced upregulation of pro-inflammatory cytokines (TNF-α, IL-1β, IL-6) was significantly inhibited in si-TMEM106A-treated macrophages.

Collectively, these data indicate that TMEM106A promotes macrophage foam cell formation, oxidative stress, and inflammatory responses, suggesting its direct involvement in AS-related macrophage dysfunction.

### 3.7 Chemokine signaling pathway enrichment and association with TMEM106A in AS

To explore the pathway-level mechanisms of TMEM106A in AS, we performed KEGG enrichment and correlation analyses using three public datasets (GSE43292, GSE100927, GSE28829).

**Figure 8:**
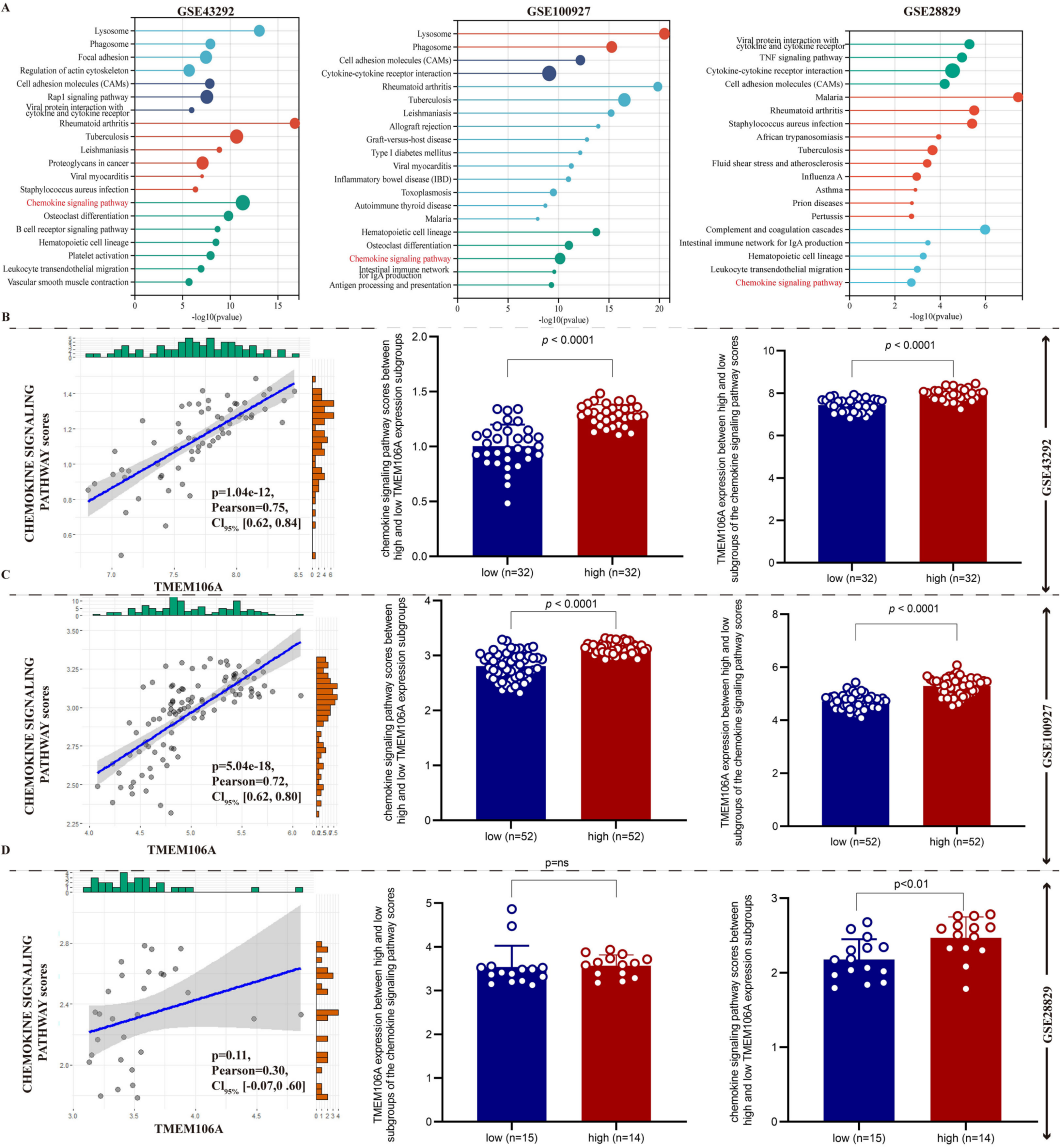
Chemokine signaling pathway and TMEM106A in AS. **(A)** KEGG enrichment of GSE43292, GSE100927, GSE28829 shows TMEM106A-related genes are significantly enriched in the chemokine signaling pathway (dots represent pathways, size indicates gene count, color reflects -log10(p-value)). **(B-D)** Correlation analyses across datasets reveal positive associations between TMEM106A expression and chemokine pathway scores; high-TMEM106A groups exhibit higher pathway activity (e.g., GSE43292: p = 1.04e-12, r = 0.75; GSE100927: p = 5.04e-18, r = 0.72; GSE28829: *p* = 0.11, r = 0.38), linking TMEM106A to chemokine pathway activation in AS.

KEGG enrichment results (**Figure 8A**) showed that in all three datasets, genes associated with TMEM106A were significantly enriched in the chemokine signaling pathway, suggesting a central role of this pathway in TMEM106A-mediated functions.

Subsequent correlation analyses (**Figures 8B–D**) revealed strong positive correlations between TMEM106A expression and chemokine signaling pathway scores across the datasets. For GSE43292 (**Figure 8B**), GSE100927 (**Figure 8C**), and GSE28829 (**Figure 8D**), both the pathway scores and TMEM106A expression levels showed significant differences between high-and low-expression groups. Specifically, higher TMEM106A expression consistently corresponded to higher chemokine signaling pathway activity, with statistical significance (p < 0.05 or lower).

These findings collectively indicate that TMEM106A is closely associated with the chemokine signaling pathway in AS, supporting the hypothesis that TMEM106A may drive AS progression by modulating chemokine-mediated immune responses.

### 3.8 TMEM106A modulates PLCB2 expression via the chemokine signaling pathway

To dissect the molecular cascade of TMEM106A in AS, we analyzed chemokine signaling-related genes across three datasets (GSE43292, GSE100927, GSE28829).

**Figure 9:**
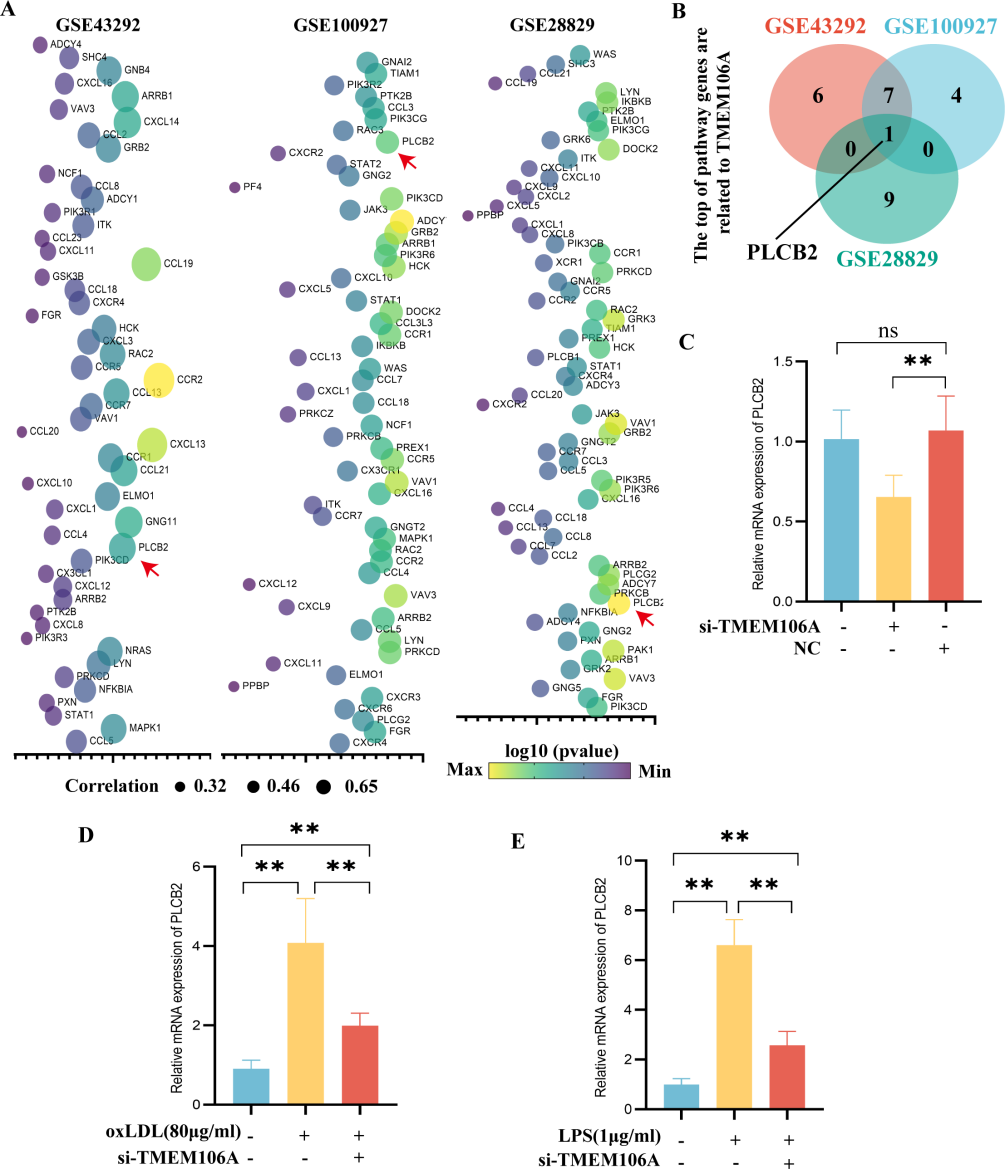
TMEM106A correlates with PLCB2 in the chemokine signaling pathway. **(A)** Correlation heatmap of TMEM106A with chemokine pathway genes across three datasets (GSE43292, GSE100927, GSE28829). **(B)** Venn diagram identifying PLCB2 as the core overlapping gene. **(C-E)** Quantitative analysis confirming si-TMEM106A inhibits PLCB2 upregulation induced by oxLDL/LPS (*p* < 0.01, n = 6). **p < 0.01.

In **Figure 9A**, correlation mapping of chemokine pathway genes with TMEM106A identified PLCB2 as a consistently significant hub gene (marked by red arrows) across all datasets. Venn diagram analysis (**Figure 9B**) further confirmed PLCB2 as the top-ranked overlapping gene in the chemokine pathway, underscoring its centrality.

To validate this, we performed RT-qPCR assays. RT-qPCR (**Figure 9C**) confirmed decreased PLCB2 mRNA expression in si-TMEM106A-treated macrophages.

Functional assays revealed that oxLDL or LPS stimulation induced PLCB2 upregulation, but this effect was significantly suppressed by TMEM106A silencing (**Figures 9D, E**). Specifically, both oxLDL-induced (**Figure 9D**) and LPS-induced (**Figure 9E**) PLCB2 elevation were attenuated in si-TMEM106A groups, as evidenced by reduced mRNA levels.

These data collectively demonstrate that TMEM106A regulates PLCB2 expression within the chemokine signaling pathway, and its silencing mitigates pro-atherosclerotic stimuli (oxLDL, LPS)-induced PLCB2 upregulation, highlighting a key molecular link in AS progression.

## 4 Discussion

Based on our experimental results, the key conclusions are as follows: First, TMEM106A is highly expressed in macrophages of atherosclerotic lesions; second, TMEM106A induces macrophage immune infiltration; third, TMEM106A upregulates chemokine expression in macrophages—collectively indicating that TMEM106A contributes to AS progression. Importantly, this evidence supports a potential regulatory role and association of TMEM106A with AS progression, rather than confirming it as a definitive driver of the disease.

TMEM106A plays an important role in immune and inflammation related diseases, mainly targeting viruses and cancer, including HIV, gastric cancer, and kidney cancer (39–41). The latest study suggests that TMEM106A can maintain partial homeostasis within macrophages (16). The above study showed that TMEM106A was significantly expressed on macrophages (42). This is consistent with our research finding that TMEM106A is correlated with biomarkers indicated by macrophages, our research indicates a positive correlation between TMEM106A and macrophage biomarkers CD68 and CD163 in the dataset. Meanwhile, our immune infiltration results indicate that TMEM106A has the highest correlation with macrophages.

Although, TMEM106A plays a role in various immune diseases, such as AIDS and cancer (39, 41). Its correlation with AS has not been previously reported. Our multi-dataset analysis, however, indicates high expression of TMEM106A in the AS group compared to the normal group. Furthermore, ROC curve analysis suggests that TMEM106A is an important potential diagnostic biomarker for AS. This was supported by the observed overexpression of TMEM106A in the aortic tissue of ApoE^-/-^ mice compared to normal mice, indicating that TMEM106A is a significant biomarker for AS.

The well-established link between AS and immunity, particularly immune cells (43), is further supported by our findings. Our GO enrichment analysis revealed a significant association between the AS dataset and immune-related diseases along with signaling pathways. By applying the Cibersort algorithm, and ssGSEA and xCell tools, we observed a strong correlation between TMEM106A expression and macrophage infiltration—echoing previous research that identified high TMEM106A expression in macrophages (42). Moreover, single-cell analysis disclosed pronounced TMEM106A expression in macrophages within AS plaques, a novel discovery corroborated by fluorescence double staining in ApoE^-/-^ mice. This suggests that TMEM106A contributes to AS progression by enhancing macrophage infiltration, potentially exacerbating the condition.

In the process of AS, chemokines play a crucial role in regulating the immune infiltration of macrophages (44). We also found a strong correlation between chemokine pathway scores and immune macrophages in three datasets. Chemokines indirectly promote the phagocytic ability of macrophages to oxLDL by promoting their migration and localization to plaque areas (45). Once macrophages aggregate and engulf oxLDL, they will further exacerbate the inflammatory response and plaque formation by releasing more chemokines and other inflammatory mediators, forming a negative cycle (46). Our enrichment analysis revealed that the genes obtained from TMEM106A differential analysis were enriched in the chemokine signaling pathway, and we validated that TMEM106A promotes the expression of chemokines. The latest research indicates that PLCB family proteins play an important role in AS. For example, PLCB3 can affect the activity of ApoE^-/-^ mice macrophages (47). Surprisingly, we found a close relationship between PLCB2 (a member of the PLCB family) and TMEM106A. In RAW264.7 cells, PLCB2 expression decreases with the silencing of TMEM106A. Therefore, TMEM106A is highly likely to inhibit the immune infiltration process of macrophages by regulating PLCB2.

Our study had several limitations that should be acknowledged. Firstly, due to the nature of public datasets used, we lacked detailed treatment information on the patients, which could potentially impact the predictive value of TMEM106A. Secondly, although we observed that TMEM106A can modulate the expression of chemokine PLCB2, further investigations are required to determine whether TMEM106A can inhibit macrophage immune infiltration through its regulation of chemokines. Lastly, it is important to note that our study relied on secondary analysis of existing public datasets, and while we validated our findings using multiple datasets and animal models, additional evidence from large-scale clinical trials is necessary to strengthen our conclusions.

## 5 Conclusion

In summary, we identified TMEM106A as an AS biomarker through multi-database mining and machine learning, elucidating that TMEM106A promotes chemokine signaling activation and macrophage immune infiltration in AS, thereby contributing to AS progression. It is explicitly clarified that the existing evidence supports a potential regulatory role and association of TMEM106A with AS progression, rather than definitive proof of causality. This work provides a potential therapeutic target for further AS research.

## Data Availability

The original contributions presented in the study are included in the article/**Supplementary Material**. Further inquiries can be directed to the corresponding authors.

## Supplementary material

Supplementary Table 1 The information of datasets.

Supplementary Figure 1 UMAP analysis and volcano map difference analysis on three datasets.

Supplementary Figure 2 ssGSEA immune infiltration analysis in the high and low TMEM106A expression groups by three datasets. **p* < 0.05, ***p*<0.01, ****p*<0.001, *****p*<0.0001.

Supplementary Figure 3 Analysis of Cibersort immune infiltration abundance in three datasets.

Supplementary Figure 4 An immune infiltration analysis was performed on high and low TMEM106A expression groups, as well as on normal and AS groups, across three independent datasets using the xCell tool. **p* < 0.05, ***p*<0.01, ****p*<0.001, *****p*<0.0001.

Supplementary Figure 5 Data processing and analysis before single-cell annotation.

Supplementary Figure 6 Flow cytometry gating strategy: Left, lymphocyte population identified by FSC-A/SSC-A; Right, FITC-A signal analysis for target cell subset (18.9% positive), ensuring valid cell selection for downstream assays.
