# Supplementary material for: TMEM106A mediates atherosclerosis progression through macrophage-centered immune responses and chemokine signaling
Source: Front Immunol. 2025 Dec 12;16:1681645. doi: 10.3389/fimmu.2025.1681645 (PMC12741138; doi:10.3389/fimmu.2025.1681645)
Supplement: Supplementary Figure 1 — UMAP analysis and volcano map difference analysis on three datasets. [file SupplementaryFile1.pdf]

TMEM106A Mediates Atherosclerosis Progression Through Macrophage-Centered Immune Responses and Chemokine Signaling

Menglong Gao[1,5], Xingbang Liu[2,5], Zhen Fang[3,5], Jia Lun[1], Qingyun Zhang[1], Yunbo Zhao[3,*],Yin Huang[4,*], Zhenhua Li[1,*]

[1]Department of Oncology, WeiFang People's Hospital, Shandong Second Medical University, Weifang, Shandong, China, 261000.
[2]Department of Cardiology, Heze Municipal Hospital，Heze, Shandong, China, 274000
[3]Department of Cardiology, WeiFang People's Hospital, Shandong Second Medical University, Weifang, Shandong, China, 261000.
[4]The Fifth Affiliated Hospital of Sun Yat-sen University
[5]These authors contributed equally.
*Correspondence: Yunbo Zhao (wfclzhaoyunbo@126.com)
Yin Huang (huangyin3@sysu.edu.cn)
Zhenhua Li (rmyylizhh@sdsmu.edu.cn)

S1

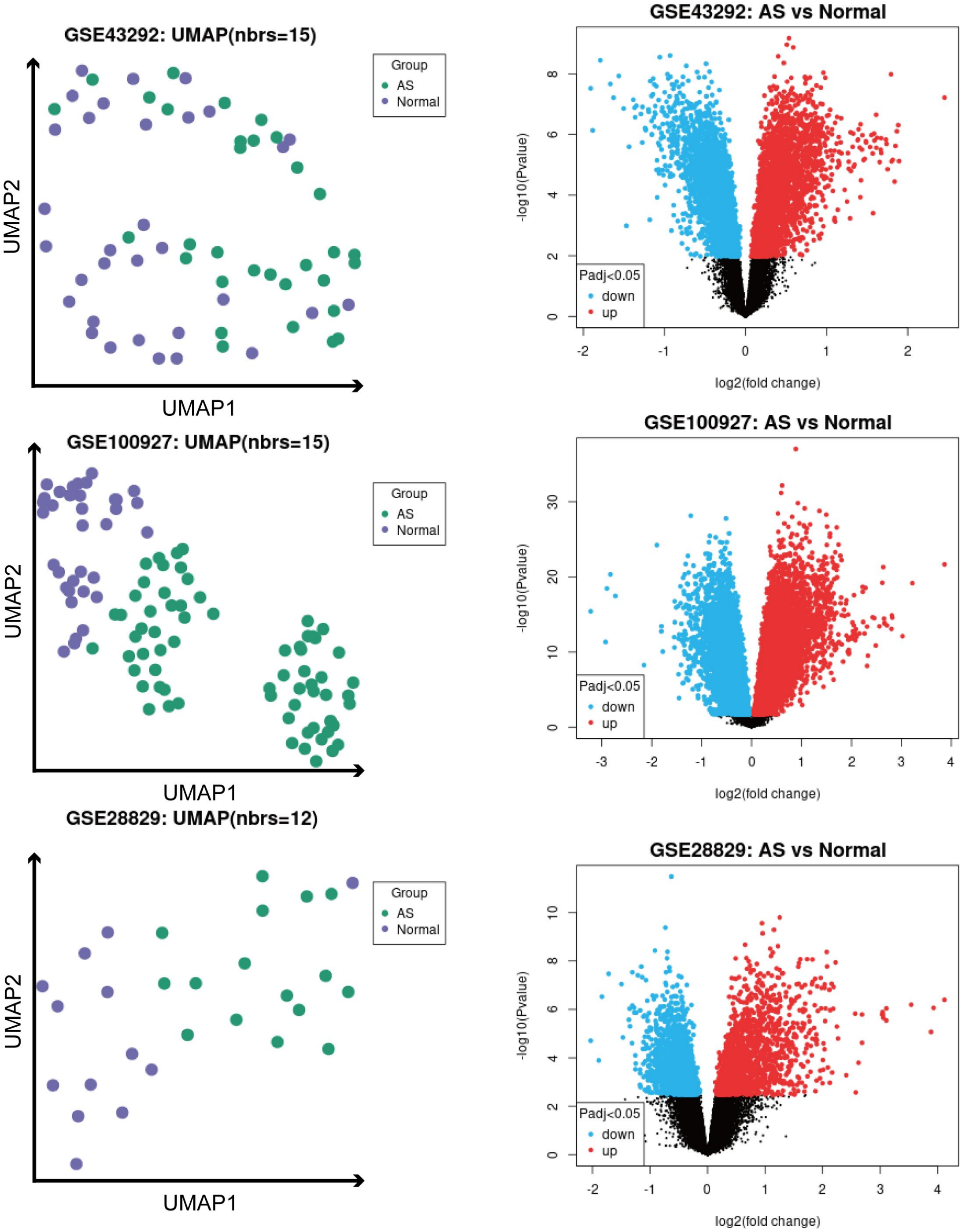

FigureS 1: UMAP analysis and volcano map difference analysis on three datasets.

S2

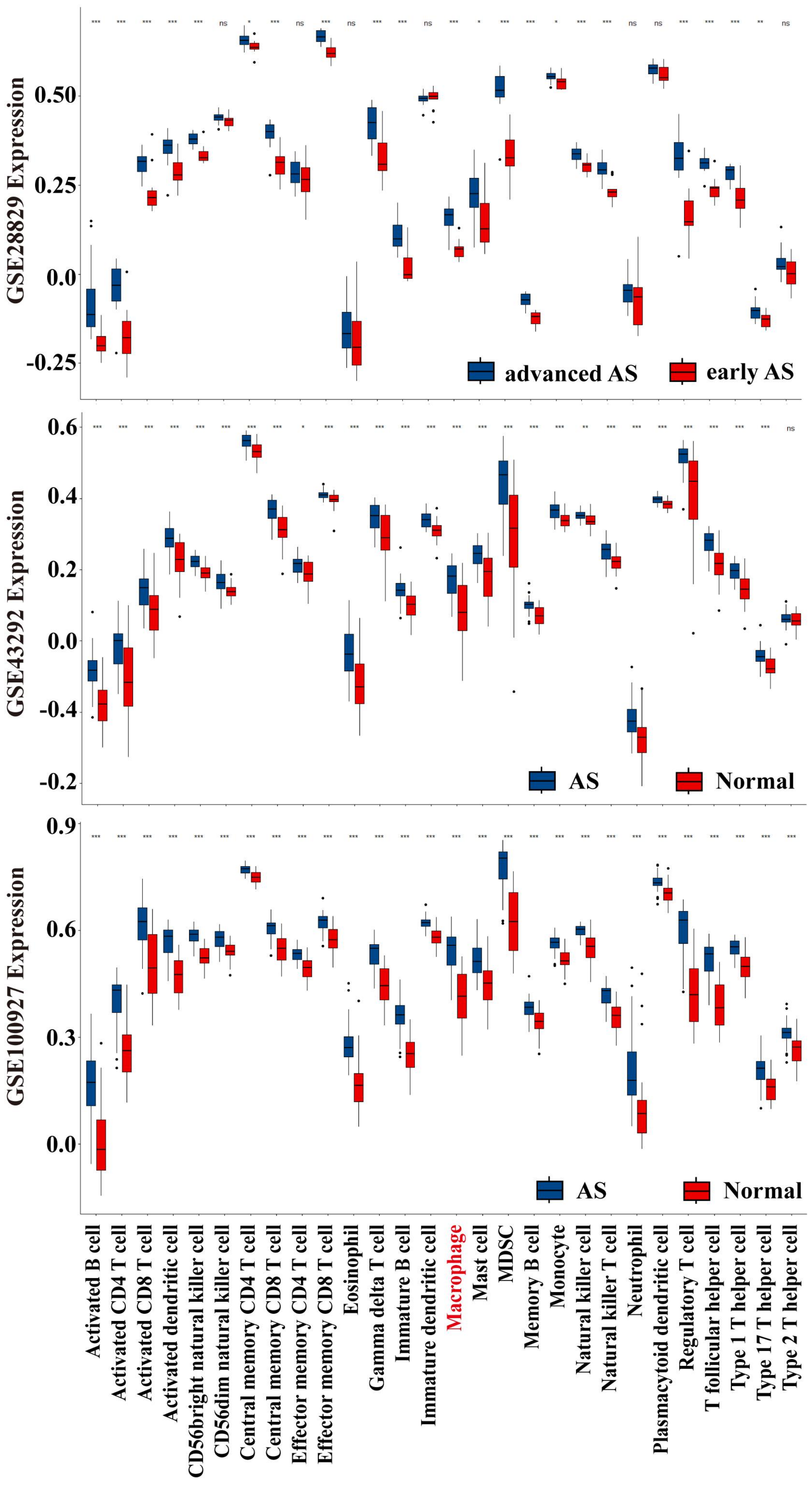
GSE28829 Expression
0.50
0.25
0.0
-0.25
advanced AS
early AS
GSE43292 Expression
0.6
0.4
0.2
0.0
-0.4
-0.2
AS
Normal
GSE100927 Expression
0.9
0.6
0.3
0.0
AS
Normal
Activated B cell
Activated CD4 T cell
Activated CD8 T cell
Activated dendritic cell
CD56bright natural killer cell
CD56dim natural killer cell
Central memory CD4 T cell
Central memory CD8 T cell
Effector memory CD4 T cell
Effector memory CD8 T cell
Eosinophil
Gamma delta T cell
Immature B cell
Immature dendritic cell
Macrophage
Mast cell
MDSC
Memory B cell
Monocyte
Natural killer cell
Natural killer T cell
Neutrophil
Plasmacytoid dendritic cell
Regulatory T cell
T follicular helper cell
Type 1 T helper cell
Type 17 T helper cell
Type 2 T helper cell

FigureS 2: ssGSEA immune infiltration analysis in the high and low TMEM106A expression groups by three datasets. *p<0.05, **p＜0.01, ***p＜0.001, ****p＜0.0001.

S3

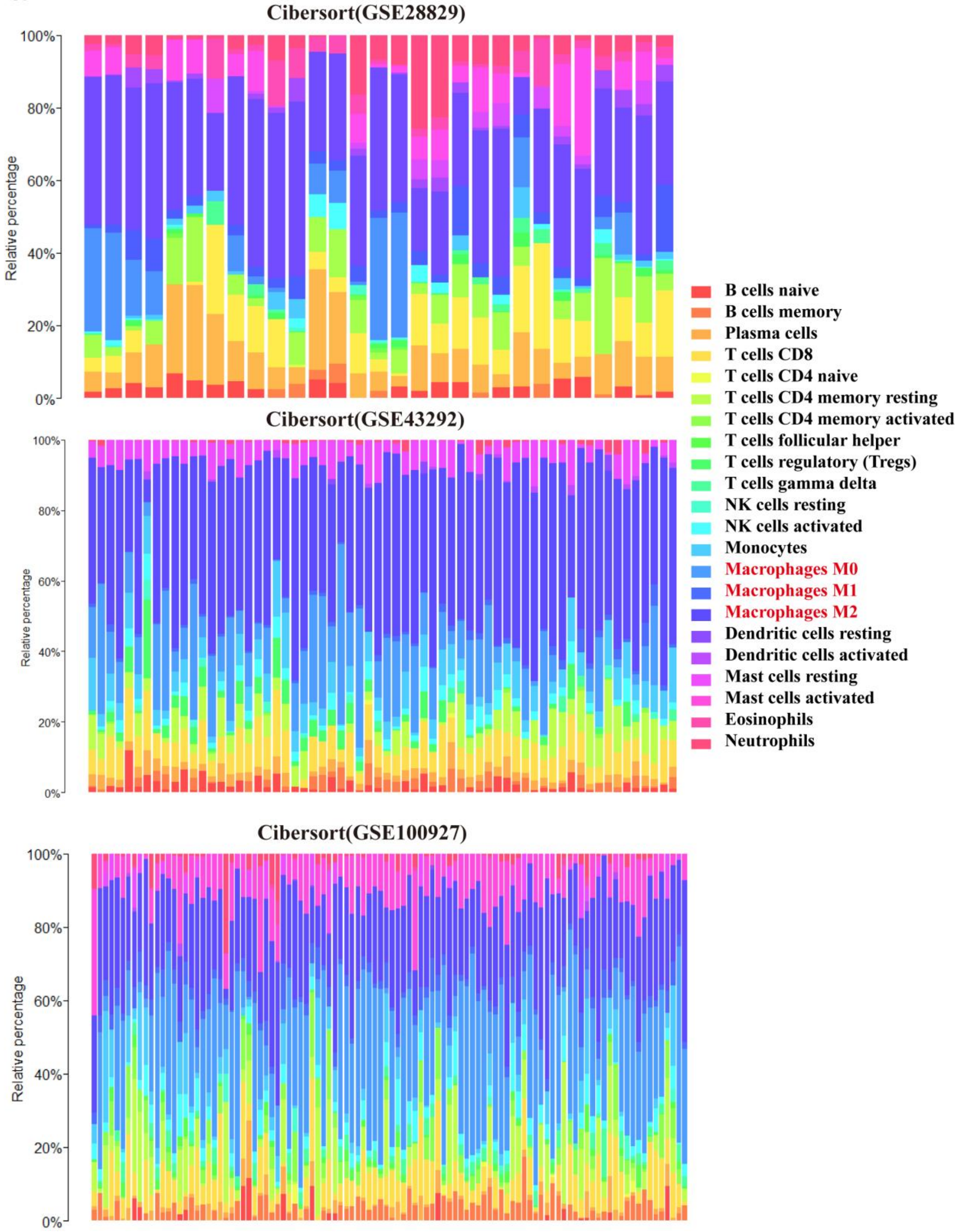

FigureS 3: Analysis of Cibersort immune infiltration abundance in three datasets.

S4

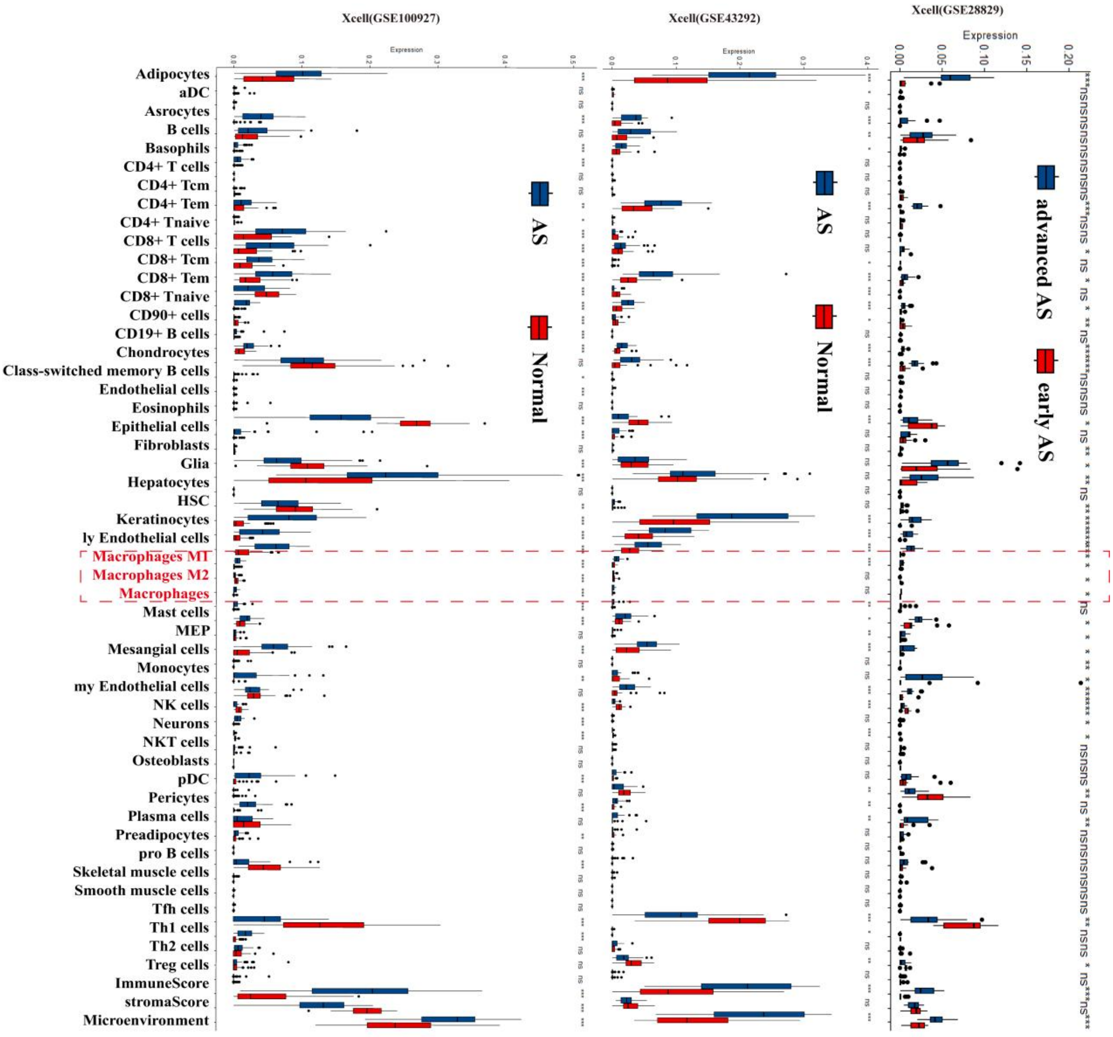

FigureS 4: An immune infiltration analysis was performed on high and low TMEM106A expression groups, as well as on normal and AS groups, across three independent datasets using the xCell tool. **p*<0.05, ***p*＜0.01, ****p*＜0.001, *****p*＜0.0001.

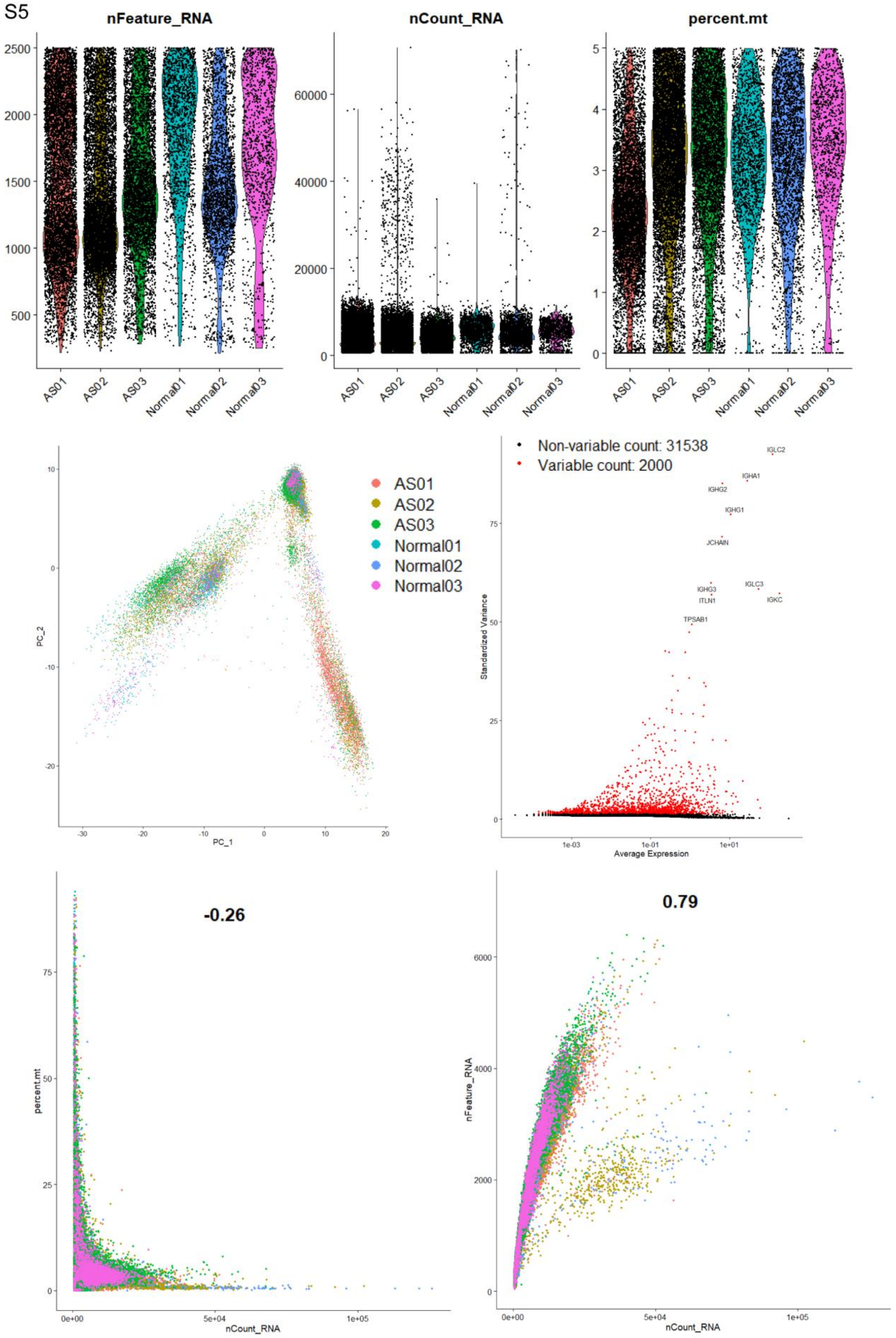

FigureS 5: Data processing and analysis before single-cell annotation.

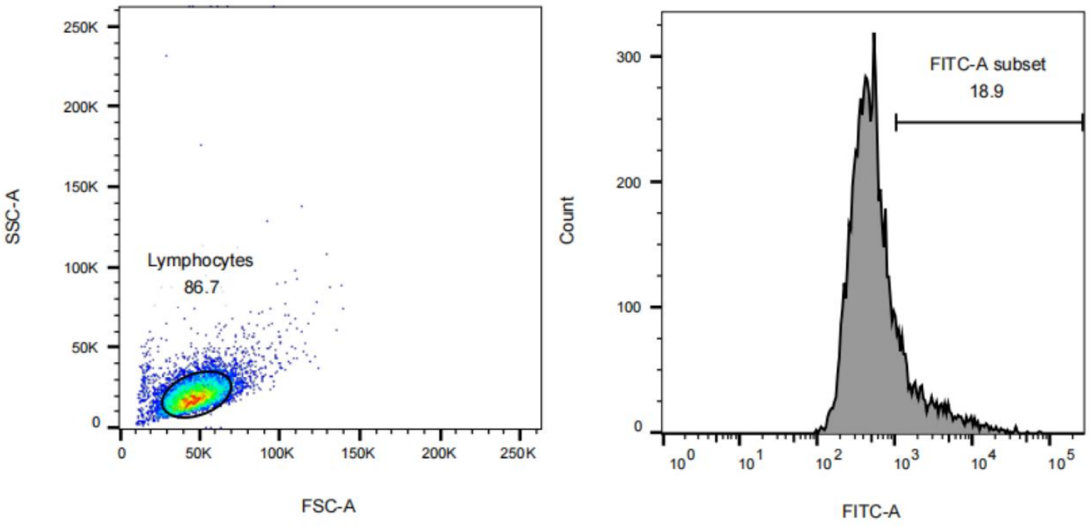

FigureS 6: Flow cytometry gating strategy: Left, lymphocyte population identified by FSC-A/SSC-A; Right, FITC-A signal analysis for target cell subset (18.9% positive), ensuring valid cell selection for downstream assays.
